# BAF200 Is Required for Heart Morphogenesis and Coronary Artery Development

**DOI:** 10.1371/journal.pone.0109493

**Published:** 2014-10-09

**Authors:** Lingjuan He, Xueying Tian, Hui Zhang, Tianyuan Hu, Xiuzhen Huang, Libo Zhang, Zhong Wang, Bin Zhou

**Affiliations:** 1 Key Laboratory of Nutrition and Metabolism, Institute for Nutritional Sciences, Shanghai Institutes for Biological Sciences, Graduate School of the Chinese Academy of Sciences, Chinese Academy of Sciences, Shanghai, China; 2 Department of Cardiac Surgery, Cardiovascular Research Center, University of Michigan, Ann Arbor, Michigan, United States of America; University of Houston, United States of America

## Abstract

ATP-dependent SWI/SNF chromatin remodeling complexes utilize ATP hydrolysis to non-covalently change nucleosome-DNA interactions and are essential in stem cell development, organogenesis, and tumorigenesis. Biochemical studies show that SWI/SNF in mammalian cells can be divided into two subcomplexes BAF and PBAF based on the subunit composition. ARID2 or BAF200 has been defined as an intrinsic subunit of PBAF complex. However, the function of BAF200 in vivo is not clear. To dissect the possible role of BAF200 in regulating embryogenesis and organ development, we generated BAF200 mutant mice and found they were embryonic lethal. BAF200 mutant embryos exhibited multiple cardiac defects including thin myocardium, ventricular septum defect, common atrioventricular valve, and double outlet right ventricle around E14.5. Moreover, we also detected reduced intramyocardial coronary arteries in BAF200 mutants, suggesting that BAF200 is required for proper migration and differentiation of subepicardial venous cells into arterial endothelial cells. Our work revealed that PBAF complex plays a critical role in heart morphogenesis and coronary artery angiogenesis.

## Introduction

Epigenetic regulation of embryonic development involves DNA methylation, histone modifications and ATP-dependent chromatin remodeling [1]. ATP-dependent chromatin remodeling complexes are specialized multi-protein machines that utilize ATP to non-covalently restructure, mobilize, or eject nucleosomes to regulate access to the DNA. One key group of this superfamily is the SWI/SNF subfamily, consisting of two closely related SWI/SNF remodeling complexes BAF (SWI/SNF-A) and PBAF (SWI/SNF-B) in mammalian cells. SWI/SNF family, as one of the most-studied chromatin remodeling complexes, plays critical roles in embryogenesis, cancer, and stem cell specification and differentiation [1]–[3].

The differential usage of the greater diversity of mammalian SWI/SNF subunits is essential for the development of specific cell fates and lineage conversion, including the progression from pluripotency to multipotency to committed cardiomyocytes and neurons [4]. For example, BAF60C is expressed specifically in the heart and somites in early mouse embryo and is essential for normal heart development by recruiting BAF chromatin remodeling complexes to heart-specific enhancers [5]. BAF53 has been implied for neuronal stem cell proliferation in mice [6], while BAF57 was reported to have important role in T-cell development in mice [7]. ATPase subunit Brg1 play an important role in heart muscle development and diseases [8], trabeculation [9] and cardiac morphogenesis and hair follicle regeneration and repair [10]. Our previous studies have shown that BAF-specific BAF250 knockout mouse embryos die early around implantation and are defective in mesodermal differentiation [11], whereas PBAF-specific BAF180 deficiency in mouse embryos leads to severe hypoplastic ventricle development and trophoblast placental defects [12], and defect in coronary vessel formation [13]. Our studies suggest that BAF and PBAF play distinct roles in vivo.

Arid2 or BAF200 has been defined as an intrinsic subunit of PBAF complex [14]. Recent study using exome sequencing of hepatocellular carcinomas revealed novel inactivating mutations of BAF200 in the liver cancer [15]. This study suggested that BAF200 could be a tumor suppressor gene that is mutated in HCV-associated hepatocellular carcinomas. In vitro functional studies showed that suppression of BAF200 by interfering RNA reduced interferon-responsive gene expression [14]. Moreover, BAF200 was required for the stability of chromatin remodeling complex. Recent work suggested BAF200 is essential for osteoblast differentiation, suggesting its role in maintaining cellular identity and activating tissue–specific gene expression [16]. However, the in vivo role of BAF200 was not known. Here we generated BAF200 knockout mice and aim to characterize its in vivo function during embryonic development.

## Materials and Methods

### Animal study

This study was carried out in strict accordance with the recommendations in the Guide for the Care and Use of Laboratory Animals of the Chinese Academy of Sciences. The protocol was approved by the Institutional Animal Care and Use Committee (IACUC) of the Institute for Nutritional Sciences, Shanghai Institutes for Biological Sciences, Chinese Academy of Sciences (Approved protocol umber 2011-AN-2). All efforts were made to minimize suffering. BAF200-LacZ mouse line was generated by knockout-first strategy by EUCOMM/Sanger Institute [17]. Mice were bred on C57BL6/J background and heterozygous male and females were mated to generate BAF200^−/−^ mutants. For proliferation study, Brdu (100 ug/g body weight) was dissolved in PBS and injected intraperitoneally into pregnant mice 4 hours before embryos harvest.

### X-gal staining

X-gal staining was performed according to previous protocol [18]. Briefly, embryonic tissues were fixed in LacZ fix solution (0.2% glutaraldehyde, 5 mM EGTA, and 100 mM MgCl_2_ in PBS) for 15–30 minutes based on the size at 4°C. After washing three times for 15 minutes in LacZ wash buffer (2 mM MgCl_2_, 0.01% sodium deoxycholate, 0.02% NP-40 in 100 mM sodium phosphate buffer), embryonic tissues were stained overnight at 37°C in LacZ stain solution (1 mg/ml 5-bromo-4-chloro-3-indolyl β-D galactopyranoside (X-gal) in LacZ wash buffer). After washing with PBS for 3 times, embryonic tissues were processed for microscopy (Leica, M165FC). For section x-gal staining, embryos and hearts were collected in PBS on ice and then fixed in 4% paraformaldehyde at 4°C for 1 hour. After washing in PBS, tissues were treated with 30% sucrose overnight. Then they were embedded in optimum cutting tissue (OCT, Sakura) and snap frozen. Cyrosections of 10 µm thickness were collected on positively charged slides. Then the section was washed by PBS for 3 times, and treated with LacZ stain solution at 37°C for 3–4 hours, after PBS wash, slides were mounted by 50% glycerol. In some samples, X-gal staining was followed by immunochemistry staining, immunochemistry staining were performed according to standard protocols after x-gal staining finished. Images were acquired on Olympus microscope BX53.

### Paraffin embedded and H&E staining

Histology was performed as previously described [19]. Mouse embryos were fixed in 4% paraformaldehyde overnight at room temperature. The following day, embryos were dehydrated through 70%, 80%, 95% and 100% ethanol for 10–30 minutes each base on the embryos' size, then embryos were moved into butyl alcohol for 2 hours and embedded with Leica paraffin. Paraffin sections (10 µm) were cut through the entire heart. Hematoxylin/eosin staining was performed on the paraffin sections. Sections were Dewaxed in xylene 2 times, and rehydrated by 100%, 95%, 80%, 75% and distilled water serially, then stained by hematoxylin solution for 4 minutes, differentiated by 1% acid alcohol for additional 1 minute, bluing in 1% ammonia water for 1 minute. Afterwards, the sections were counterstained by eosin Y solution for 10 seconds, and dehydrated by serial ethanol and xylene.

### Immunohistochemistry

Immunostaining was performed according to protocols described previously [20]. Briefly, embryos were collected in PBS on ice and then fixed in 4% paraformaldehyde at 4°C for 1 hour. After washing in PBS, tissues were treated with 30% sucrose overnight. Then they were embedded in optimum cutting tissue (OCT, Sakura) and snap frozen. Cyrosections of 10 µm thickness were collected on positively charged slides. Tissues were blocked with PBS supplemented with 0.1% Triton X-100 and 5% normal donkey serum (Jackson ImmunoResearch) for 1 hour at room temperature, followed by first antibody incubation overnight at 4°C. Signals were developed with Alexa fluorescence-conjugated antibodies (Invitrogen). For weak signals, we used HRP or biotin-conjugated antibodies with tyramide signal amplification kit (PerkinElmer). Immunofluorescent detection of BRDU (Abcam, AB6326, 1∶100), ACTN2 (Sigma, A7811, 1∶100), P57^kip2^ (Thermo Scientific, MS-897-P0, 1∶50), phosphorylated histone H3 (Upstate, 06-570, 1∶1000), MYL7 (Santa Cruz, SC-66967, 1∶50), MYL2 (Sigma, HPA019763, 1∶50), GATA4 (R&D, AF2606, 1: 100), NKX2-5 (Santa Cruz, SC-8697X, 1∶100), PECAM (BD Pharmingen, 553370, 1∶500), AP2 (Abcam, AB13979, 1∶500), LYVE1 (Abcam, AB14917, 1∶100), TNNI3 (Abcam, AB56357, 1∶200) was performed on cyrosections. Images were acquired on Olympus confocal microscope FV1000, Zeiss confocal microscope LSM510 and Olympus microscope BX53. Quantification was performed by a blinded observer to experiment.

### TUNEL staining

TUNEL staining was performed using *In Situ* Cell Death Detection Kit, TMR Red (Roche, 12156792910). Frozen tissue sections were fixed with 4% paraformaldehyde for 10–20 minutes at room temperature, and washed 30 minutes with PBS, then moved slides into PBST (0.1% Triton X-100 in PBS) for 2 minutes at 4°C. Slides were incubated with TUNEL reaction mixture for 1 hour at 37°C, wash with PBS 3 times. Afterwards, tissue sections were then counter-stained with cardiomyocyte marker ACTN2. Images were acquired on fluorescence microscope (Olympus, BX53).

### Whole-mount PECAM staining

Whole-mount PECAM staining was performed as previously described [18]. Briefly, mouse embryos were collected in PBS on ice and fixed in 4% paraformaldehyde overnight at 4°C. The next day, embryos were washed in PBS three times, followed by serial dehydration by methanol. Then embryos were bleached in 5% hydrogen peroxide in 100% methanol for 2 hours at 4°C, followed by rehydration. Embryos were blocked in PBS containing 5% normal donkey serum and 0.1% Triton X-100 for 1 hour at 4°C. Embryos were then incubated in block solution containing PECAM antibody (BD PHharmingen, 553370) overnight at 4°C, followed by five times wash in PBS. Then, embryos were incubated with ImmPRESS Anti-Rat Ig (Vector Lab) for 2 hours at room temperature and washed in PBS for five times, then developed by DAB (Vector Lab) at room temperature.

### Explant assay

A solution (1 mg/ml) of collagen type I (BD Biosciences 354236) was dispended into 4-well microculture dishes (NUNC) and allowed to solidify inside a 37°C, 5% CO_2_ incubator. Collagen gels were washed several times with Opti-MEM. Subsequently, the wells were filled with Opti-MEM containing 1% rat serum, 1% insulin-transferrin-seleniun (ITS, Invitrogen) and antibiotics, and incubated overnight. heart explants were harvested in sterile PBS from E12.5 embryos. Explants were placed with the epicardium facing downwards and allowed to attach for 5 hours at 37°C, 5%CO_2_. DMEM containing 10% FBS and antibiotics was added and the cultures were incubated for up to 3 days.

### Quantitative RT-PCR analysis

**Figure 1:**
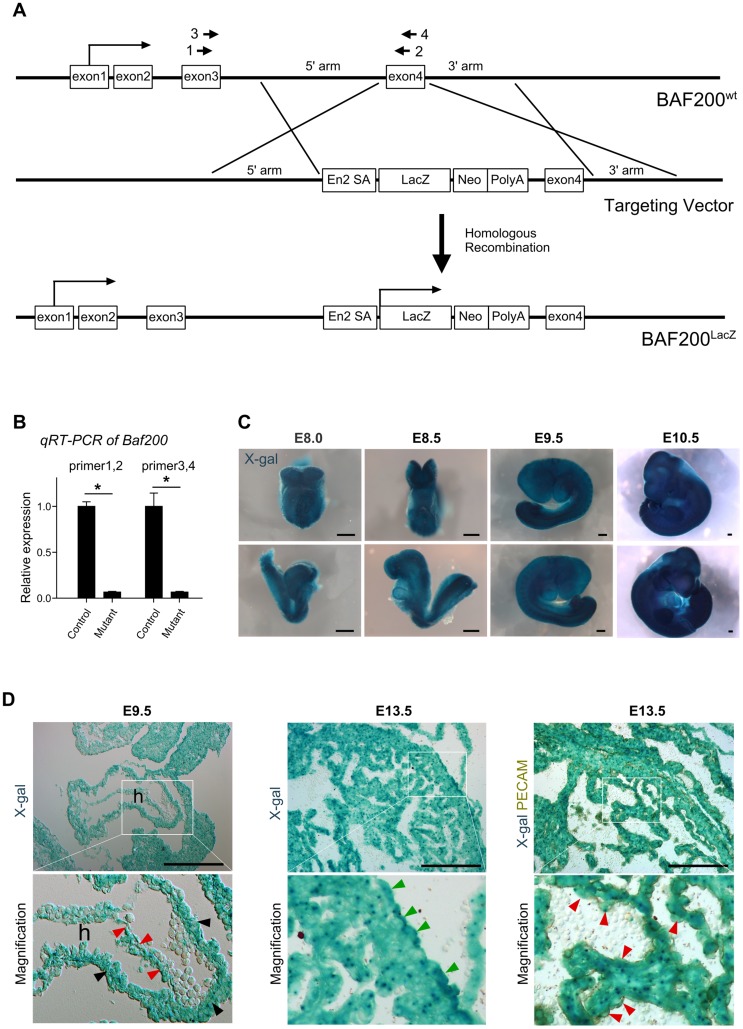
Generation of BAF200 knockout mouse. (**A**) LacZ cDNA was targeted into BAF200 gene locus by homologous recombination, generating early termination of BAF200 transcription. 1–4 indicates four different primers that span exon 3 and exon 4. (**B**) Quantitative RT-PCR shows significantly reduced *Baf200* transcripts in BAF200^LacZ/LacZ^ mutants compared with littermate control. n = 8. *P<0.05. (**C**) Whole mount x-gal staining of BAF200^LacZ/+^ at E8.0 to E9.5. White Representative of 3 for each time point. (**D**) X-gal staining of E9.5 and E13.5 heart section. h, heart; red arrowheads point to endocardial cells; black arrowheads indicate cardiomyocytes in compact myocardium; green arrowheads indicate epicardial cells. Bar  = 200 µm.

Mice hearts were collected from E13.5 embryos, RNA was extracted with Trizol according to manufacturer's protocol (Invitrogen) and converted the RNA to cDNA using M-MLV reverse transcriptase (Promega, M170A). For qPCR, SYBR Green qPCR master mix (Applied Biosystems) was used and cDNA was amplified on a Applied Biosystems® 7500 Real-Time PCR System. qRT-PCR primers for BAF200 is designed to span exon 3 and exon 4 (Figure 1A): Primer1 (exon3): TGTTCCAACGCTGCCTTTGC; primer 2 (exon4): TGGCTTTGGATTGCCTGGTG; primer 3 (exon3):AGAAGTTGTTCCAACGCTGCC; Primer 4 (exon4): TGTGGCTTTGGATTGCCTGG; Fabp4 qRT-PCR primers: (F):CACCATCCGGTCAGAGAGTA; (R):TGATGCTCTTCACCTTCCTG.

### Statistics

Data were analyzed by unpaired student's-t-tests for two groups. Significance was accepted when *P*<0.05. All data are presented as mean ±SEM.

## Results and Discussion

**Figure 2:**
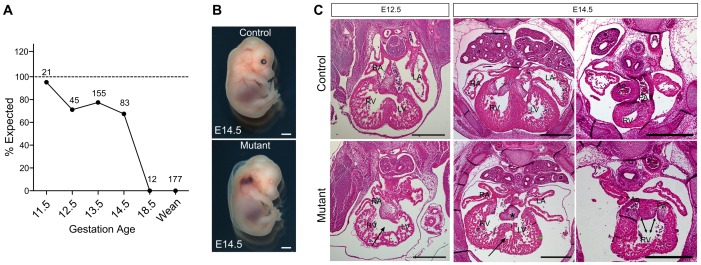
Survival of offspring and heart defects in BAF200 mutant. (**A**) The graph displays the frequency of offspring of BAF200 mutants. The number of offspring genotyped for each time point is indicated. (**B**) Whole mount view of mutant and littermate control embryos. White bar  = 1 mm. (**C**) H.E. staining of E12.5 and E14.5 BAF200 mutant and littermate control embryos show thin compact myocardium (green bar), common atrioventricular valve (asterisks), ventricular septum defect (arrows) and double outlet right ventricle (DORV, double arrows). Black bar  = 0.5 mm.

BAF200-LacZ knockin mouse line was generated by knockout-first strategy [17]. LacZ cDNA was inserted into BAF200 locus, potentially causing an early termination of transcription of BAF200 gene (Fig. 1A). We measured the remaining *Baf200* transcripts in BAF200^LacZ/LacZ^ mutant embryonic samples by quantitative reverse transcriptase-polymerase chain reaction (qRT-PCR), and found significant reduction of *Baf200* RNA expression in mutants compared with littermate controls (Fig. 1B). Since there was a trace amount of Baf200 transcripts (∼7%), this BAF200 mutant allele is considered as hypomorph. Whole mount X-gal staining of BAF200^LacZ/+^ embryos showed that BAF200 was expressed in most tissues at early stages (Fig. 1C). Sectional X-gal staining showed its expression in epicardium, endocardium and myocardium in developing heart (Fig. 1D). We next used this BAF200 LacZ allele to examine its in vivo function during embryonic development. Intercrosses between BAF200 heterozygous mice did not produce any live BAF200 homozygous mutant offspring (0/177), and analysis of litters from timed mating showed that BAF200 mutant embryos died between E12.5-E14.5 (Fig. 2A). These data indicated that BAF200 is required for normal embryonic development. By focusing on embryonic stages, we found that BAF200 mutant embryos developed subcutaneous edema and hemorrhage (Fig. 2B), suggesting severe impairment of cardiac function. We therefore performed histological examination of E12.5 to E14.5 mutants. By H.E. staining, we found that BAF200 mutant had a constellation of cardiac defects that included thin compact myocardium, common atrioventricular valve or atrioventricular valve defect, ventricular septum defect, double outlet right ventricle (Fig. 2C). These phenotypes resembled some of the most common forms of congenital heart defects, suggesting that dysfunction of BAF200 may lead to congenital heart diseases.

**Figure 3:**
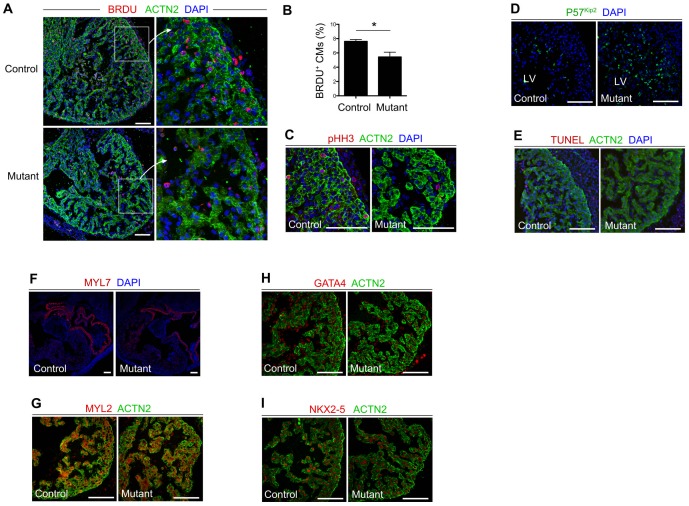
Reduced cardiomyocyte proliferation in BAF200 mutant hearts. (**A, B**) Staining of BRDU shows reduced cardiomyocytes (CMs) proliferation in E13.5 BAF200 mutant hearts compared with littermate controls. *P<0.05. (**C**) phospho-histone H3 staining shows reduced proliferation in BAF200 mutant compact myocardium. (**D**) Expression of p57kip2, a cyclin-dependent kinase inhibitor, is increased in mutant hearts. (**E**) No significant apoptosis was detected in mutant and control embryos. (**F, G**) Expression of chamber-specific markers MYL2 and MYL7 was unchanged in BAF200-deficient hearts. (**H, I**) Expression of cardiac transcription factors GATA4 and Nkx2-5 were not significantly changed in BAF200 mutants. White bar  = 100 um.

The thin compact myocardium was prominent among all samples, so we next examined the proliferation and differentiation of cardiac tissues in the mutant hearts. BRDU labeling indicated that the number of proliferating cardiomyocytes in BAF200 mutants was significantly reduced compared with that of littermate controls (Fig. 3A, B). Cell proliferation defect was confirmed by another marker phospho-histone H3 staining (Fig. 3C). Examination of a cyclin-dependent kinase inhibitor, p57^kip2^ was dramatically increased in mutant (Fig. 3D). However, we did not observe significant increase of cell death in mutants compared with control littermates (Fig. 3E). Cardiac differentiation markers MYL2 and MYL7 were not significantly changed in mutant, suggesting normal differentiation of cardiac chamber in BAF200 mutant hearts (Fig. 3F, G). Furthermore, GATA4 and NKX2-5, two key cardiac transcription factors [21], [22], were normally expressed in BAF200 mutant hearts (Fig. 3H, I). Altogether, these data suggest that BAF200 is required for normal heart development, and regulates cardiomyocyte proliferation.

**Figure 4:**
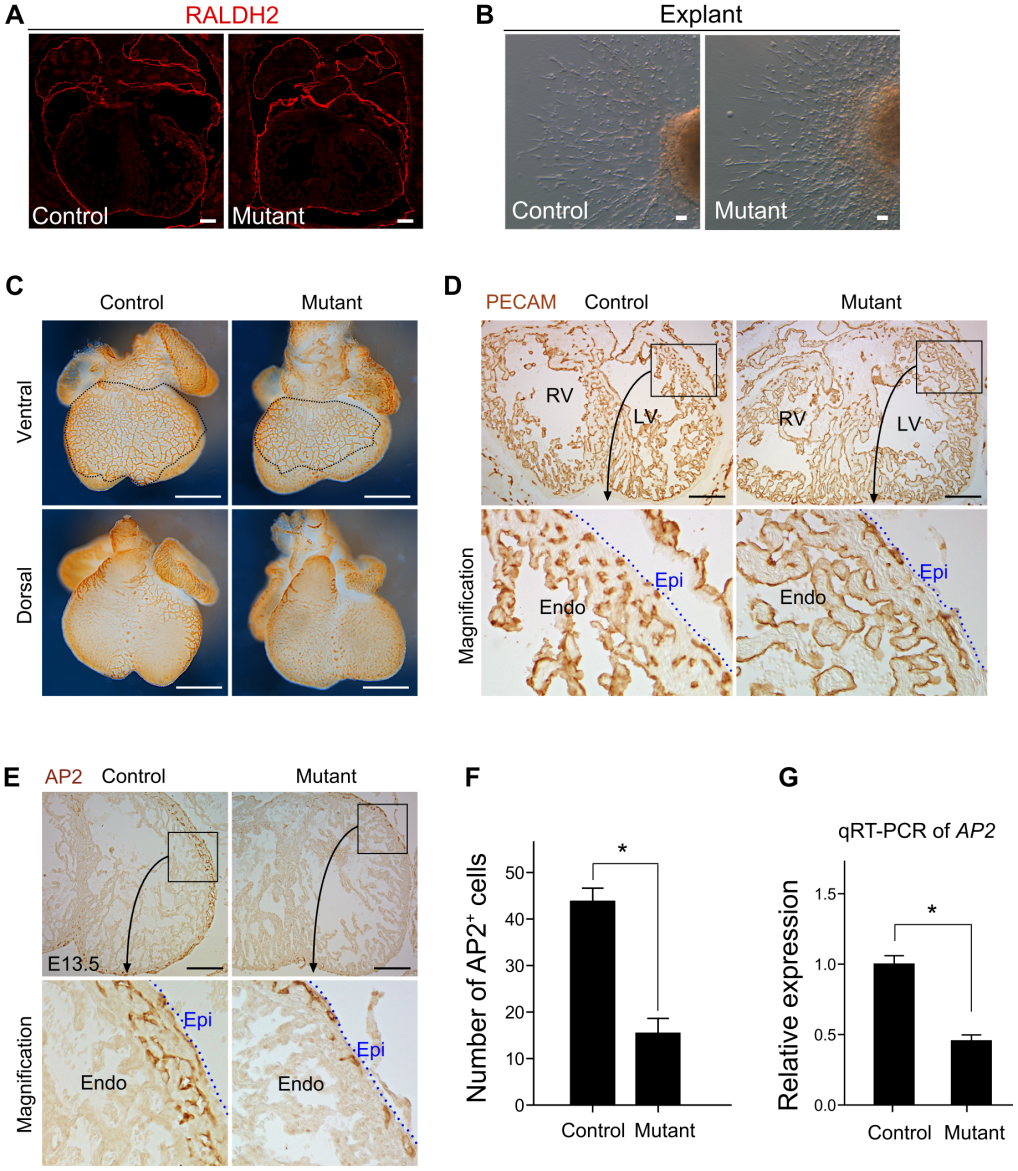
Coronary angiogenesis was impaired in BAF200 mutant hearts. (**A**) Epicardium marker RALDH2 indicates that epicardium integrity remains in E13.5 BAF200 mutants. (**B**) E12.5 heart explant assays measuring migrating epicardial cells in vitro showed no EMT defect in mutant hearts. White bar  = 100 um. (**C**) Whole mount PECAM staining of E13.0 embryonic hearts. White bar  = 0.5 mm. (**D**) Immunostaining of PECAM on E13.5 embryonic hearts. Dotted blue lines indicate epicardium (Epi). Endo, endocardium; LV, left ventricle; RV, right ventricle. Black bar  = 0.2 mm. (**E**) Immunostaining of AP2 on E13.5 hearts shows vascular endothelial cell (EC) patterning in embryonic hearts. Black bar  = 0.2 mm. (**F**) Quantification of intramyocardial ECs in BAF200 mutant and littermate control hearts. *P<0.05; n = 3. (**G**) Quantitative RT-PCR (qRT-PCR) of vascular endothelial cell marker *AP2*. *P<0.05; n = 8.

We next attempted to dissect the possible function of BAF200 in epicardium development and coronary formation, as BAF180 is involved in these processes [13]. The heart is avascular at E10.5, and coronary vessels begin to form afterwards when compact myocardium become thicker. Normal formation of epicardium is crucial for early coronary vascular development [23], [24]. Staining of epicardial marker RALDH2 showed that epicardium was formed normally in mutant hearts (Fig. 4A). In addition, Ex vivo explant assays showed no difference in migrating epicardial cells number and epithelial-to-mesenchymal transition (EMT) between BAF200 mutants and littermate controls (Fig. 4B). We next examined the coronary vasculature in BAF200 mutant hearts. By whole mount PECAM staining, we found that subepicardial coronary vasculature was in similar pattern to littermate controls (Fig. 4C). However, sectional staining of PECAM revealed that intramyocardial coronary vascular endothelial cells (ECs) were significantly reduced in BAF200 mutants (Fig. 4D). To identify the vascular defects more specifically, we applied immunostaining of the coronary vascular specific marker AP2 [25]. Intramyocardial coronary arteries were significantly reduced in BAF200 mutants (Fig. 4E, F). This was also confirmed by quantitative RT-PCR (Fig. 4G). These coronary arteries were proposed to be formed by reprogramming of subepicardial coronary venous cells [26], [27]. Since intramyocardial coronary vessels contain most coronary artery endothelial cells while subepicardial endothelial cells will become coronary veins, specific reduction in coronary arterial endothelial cells instead of venous population suggested that BAF200 regulated coronary artery differentiation and formation. Further study of BAF200 in regulation of coronary vessel formation would aid in our understanding of reprogramming of vascular cells and may provide new avenues for vascular regeneration following injury and diseases [28].

**Figure 5:**
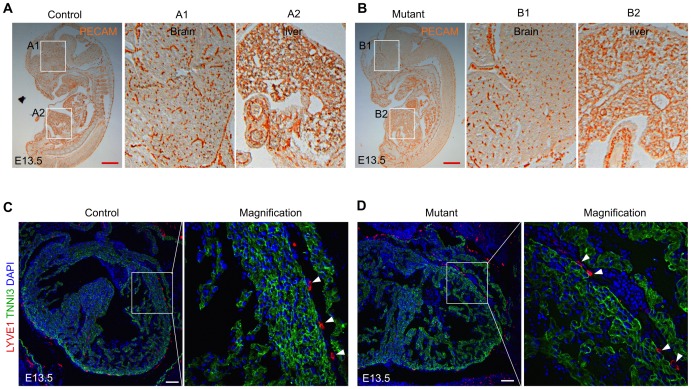
Cardiac lymphatic vessels and vessels in other organs are not impaired in BAF200 mutants. (**A, B**) Immunostaining of PECAM on E13.5 control and mutant embryos. A1, A2, B1, B2 are magnified pictures showing vessels in brain and liver. Representative figure of 3 samples. Red bar  = 1 mm. (**C, D**) Immunostaining of LYVE1, TNNI3 and DAPI on E13.5 control and mutant hearts. LYVE1^+^ lymphatic vessels (white arrowheads) are not impaired in mutants compared with littermate controls. White bar  = 100 µm.

We next determined if the defects of angiogenesis in the BAF200 mutants were cardiac coronary vessel-specific or the result of a general angiogenesis defect, giving the fact that BAF200 is widely expressed throughout the whole embryos (Fig. 1C). We performed PECAM immunostaining on E13.5 BAF200 mutants and littermate controls, and did not find any significant defect in vessel development in other organs or tissues eg. brain, liver (Figure 5A and 5B). We therefore conclude that coronary vessel defect is cardiac-specific. To test if other type of vessels like cardiac lymphatic vessels are impaired in growth, we performed immunostaining of lymphatic vessel marker LYVE1 on BAF200 mutants. We found there was no significant defect in LYVE1^+^ number in BAF200 mutants compared with littermate controls (Figure 5C and 5D).

Our study provided the first in vivo evidence that BAF200 played important roles in embryonic cardiomyocyte proliferation as well as in lineage conversion of venous cells into arterial endothelial cells during coronary development. Interestingly, this data recapitulated the phenotype of BAF180 mutants [13], [14], suggesting that the observed functions of BAF200 is PBAF-specific. Our studies may reveal new clues into the etiology of various congenital heart diseases. Moreover, understanding the cardiomyocyte proliferation and coronary reprogramming processes governed by SWI/SNF complexes and identifying the endogenous regulators should provide novel insights into strategies of repopulating and revascularizing the cardiac tissue after myocardial infarction or other ischemic diseases.
